# Physicochemical Properties of Biopolymer Hydrogels Treated by Direct Electric Current

**DOI:** 10.3390/polym8070248

**Published:** 2016-07-12

**Authors:** Żaneta Król, Magdalena Malik, Krzysztof Marycz, Andrzej Jarmoluk

**Affiliations:** 1 Department of Animal Products Technology and Quality Management, Wroclaw University of Environmental and Life Sciences, Chelmonskiego 37/41, 51-630 Wroclaw, Poland; andrzej.jarmoluk@up.wroc.pl; 2 Faculty of Chemistry, Wroclaw University of Technology, Smoluchowskiego 23, 50-370 Wroclaw, Poland; magdalena.malik@pwr.edu.pl; 3 Department of Environment Hygiene and Animal Welfare, The Faculty of Biology and Animal Science, Wrocław University of Environmental and Life Sciences, Chelmonskiego 38 C, 50-630 Wrocław, Poland; krzysztof.marycz@up.wroc.pl

**Keywords:** gelatine, carrageenan, sodium alginate, hydrogel, direct current, swelling ratio, TPA, SEM, FTIR

## Abstract

The objective of this study was to evaluate the changes within the physicochemical properties of gelatine (2%; 4%; 8%), carrageenan (1.5%; 2%; 2.5%) and sodium alginate (0.75%; 1%; 1.25%) hydrogels with different sodium chloride concentrations that were triggered by applying direct current (DC) of 400 mA for a duration of five minutes. There were three types of gels prepared for the purpose of the study: C, control; H, gels on the basis of hydrosols that were treated with DC; and G, gels treated with DC. In the course of the study, the authors carried out the following analyses: Texture Profile Analysis (TPA), Fourier Transform Infrared spectroscopy (FTIR), Scanning Electron Microscopy (SEM) and Swelling Ratio (SR). Furthermore, the color and pH of hydrogels were measured. The FTIR spectra showed that the structures of gelatine, carrageenan and sodium alginate do not significantly change upon applying DC. The results of TPA, SR, color and pH measurement indicate that hydrogels’ properties are significantly dependent on the type of polymer, its concentration and the type of the gel. By changing those parameters, the characteristics of such gels can be additionally tuned, which extends their applicability, e.g., in the food industry. Moreover, the analysis revealed that SR of H gel gelatine after 72 h of storage was 1.84-times higher than SR of the control sample, which indicated that this gel may be considered as a possible component for wound dressing materials.

## 1. Introduction

Hydrogels are materials composed of a polymer backbone, water and, potentially, also a crosslinking agent. Due to their hydrophilic nature and three-dimensional polymeric network, hydrogels can absorb water or biological fluids [1]. Hydrogels can also exhibit phase transition (i.e., volume change) in response to changes in external conditions, such as pH, ionic strength, temperature and electric current. These materials are known as the “stimuli-responsive” or “intelligent/smart” hydrogels [2]. Hydrogels also offer the possibility of fabrication in a variety of different shapes, e.g., films, discs, rods and microparticles, which results in a variety of applications in the medical and pharmaceutical fields and other related industries [3,4]; additionally, they also may find use in food production [5].

Gelatine is a denatured fibrous protein obtained from collagen by a controlled partial thermal hydrolysis [6]. Gelatine solution forms a reversible hydrogel. This change is governed by the physical interactions amongst the molecules [7]. One such interaction is the electrostatic activity in the presence of electric charges on the polypeptide chain. Another is the formation of the hydrogen bond between the amino acid units, which participate in gel formation [8,9]. Gelatine with high levels of hydroxyproline tends to have high gel properties [10]. The hydroxyl groups of hydroxyproline play a part in the stability of the helix by inter-chain hydrogen bonding via bridging water molecules, as well as direct hydrogen bonding to the carbonyl group [11]. The properties of gelatine gels may depend on pH and gelatine concentration. At low polymer concentration, three regions of the helix may be derived from one chain to give an intramolecular collagen-like structure, which makes no contribution to the gel network. At higher gelatine concentrations, the three regions of the helix can come from two or three different chains, so that useful junction zones that induce gelation can be formed. Moreover, gelation of both gelatine types A and B is low outside the pH range 4.0–10. This is attributed to strong electrostatic forces that inhibit the ability of chains to form junction zones [12].

Gelatine is used to produce hydrogels owing to its qualities, such as non-toxicity, high water absorption, biodegradability and biocompatibility. These features make gelatine an excellent material for drug delivery systems [13], wound dressing applications [14,15,16] and films and coatings for food applications [17,18].

Carrageenan is an assortment of sulfated polyelectrolyte heteropolysaccharides extracted from certain species of red seaweed (*Rhodophyta*) [19,20,21]. Its chemical structure consists of a chain of alternating β-d-galactose and α-d-galactose linked together through β-(1,4) and α-(1,3) links. There are three main types of carrageenan (κ, ι, λ-carrageenans) that differ from one another according to their 3,6-anhydrogalactose content and the number of sulfate groups present in the structure along the chain [22]. Carrageenan forms a thermoreversible gel as a function of temperature and ionic concentration [23]. Within the carrageenan family, κ-carrageenan originates the strongest gels [24]. Carrageenan is widely used in food production as a thickening, gelling and stabilizing protein-suspending agent. It can also be utilized in non-food industries as excipients in pharmaceutical pills and tablets or in the immobilization of biocatalysts [25].

Alginate is a natural anionic polyelectrolyte extracted from various species of brown algae (*Phaeophyceae*) and bacteria (*Azotobacter vinelandii*). It is composed of 1-4 linked α-l-guluronic and β-d-mannuronic acid residues [26]. Alginate is known to form gel in the presence of divalent cations, such as Ca^2+^, which act as cross linkers between the functional groups of alginate chains [27]. Alginate is considered biocompatible, biodegradable and non-toxic, and its use as a food additive has been Generally Recognized As Safe (GRAS) by the Food and Drug Administration (FDA) since 1982 (21CFR184.1724) [28].

In recent years, researchers have investigated the possibility of using Direct electric Current (DC) in hydrogels for electrically-stimulated drug delivery [29,30,31,32]. However, there are no studies on the possibility of using hydrosols/hydrogels treated with DC (≥400 mA) as a form of edible food components with antimicrobial properties. As microbial spoilage is a major cause of food deterioration and food borne diseases are significant causes of morbidity and economic loss, the application of antimicrobial coating or food components is a very promising research area [33,34]. Our previous study revealed that using a weak DC (10–30 mA) for a short time on inoculated agar plates inhibits the growth of *S. aureus* and *Y. enterocolitica*, and the inhibitory effect can be enhanced upon the addition of sodium chloride [35]. Therefore, the results have shown that direct usage of DC can be a novel method of non-thermal sterilization. However, the applicability of our method can be extended by using higher amperage. After applying weak DC, there was no Available Chlorine Concentration (ACC) that exhibits antibacterial activity [36,37] in hydrosols treated with DC, whereas after applying higher amperage (200–400 mA), ACC was present [38]. The presence of ACC provides antimicrobial activity sustained upon storage. This means that the use of DC is not the only method for sterilization, but also, it allows extending the shelf life and reducing or eliminating the addition of preservatives. Therefore, the authors believe that the use of a DC flow in the hydrosol/hydrogel layer can be used for new active packaging material preparation or edible food components, which would exhibit antibacterial qualities.

The study aimed to evaluate the changes in the physiochemical and mechanical properties of gelatine, carrageenan and sodium alginate hydrogels after the application of DC.

## 2. Experimental Section

### 2.1. Apparatus

**Figure 1:**
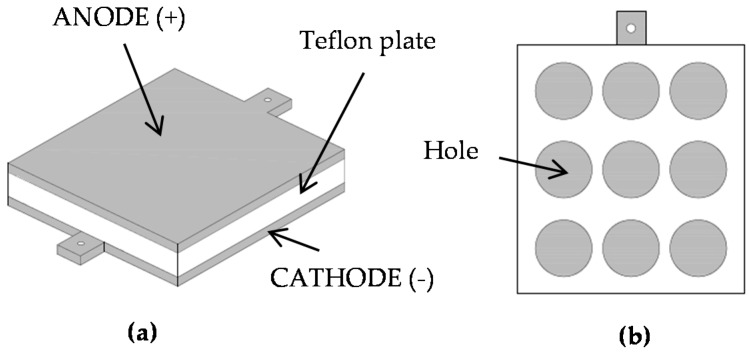
Schematic of the experimental set-up employed for the DC treatment of hydrosols and hydrogels; (**a**) side view, (**b**) cross-section.

The apparatus used to treat the samples with DC is presented in Figure 1. The hydrosols/hydrogels were placed into holes in a Teflon plate. The graphite electrodes were kept in contact with the opposite surface of the hydrosol/hydrogel. The electric current was provided from a DC power supply, Major Science MP-SAP (Major Science, Saratoga, CA, USA). During all of the experiments, the samples were treated with DC of 400 mA for 5 min. The controls were treated in exactly the same manner as the research sample, except that no electric current was applied.

### 2.2. Materials

Gelatine from porcine skin (180 Bloom) was purchased from Weishardt (Graulhet, France), and κ-carrageenan was acquired from Regis (Kraków, Poland). Alginate FD 125 was obtained from Dupont GRINSTED^®^, Grindsted, Denmark.

### 2.3. Preparation of Hydrogels

The gelatine/carrageenan/sodium alginate was dissolved in distilled water containing NaCl. The composition of the obtained solutions is shown in Table 1. The gelatine and carrageenan sols were heated to 60 °C and stirred continuously (IKA, RW 20 digital) at 300 rpm for 10 min, while the sodium alginate sols were stirred for 30 min at room temperature.

The gelatine and carrageenan hydrosols were poured into holes in the Teflon plate (Figure 1). The graphite electrodes were put on the opposite surface of the plates. There were three types of gels prepared (Table 2). The gels were formed in a refrigerator (3 °C) for 24 h. After removal from the plate, cylinder-shaped hydrogel blocks with a base diameter of 25 mm and a height of 20 mm were obtained.

The control (C) gels from sodium alginate (Table 2) were prepared by pouring hydrosols into a semi-permeable cellulose casing. The gels on the basis of hydrosols (H) were prepared by pouring hydrosols from the Teflon plate into a semi-permeable cellulose casing. The obtained bars were immersed in 0.5 M CaCl_2_ solution for 24 h, and then, they were cut into blocks with a base diameter of 25 mm and a height of 20 mm. In order to receive the gels treated with DC (G) samples, blocks were placed in the Teflon plate holes and treated with DC.

### 2.4. Hydrogels’ Characterization

#### 2.4.1. pH Measurement

The pH was measured in the geometric centers of the gels using an electrode (Hanna instruments, Ann Arbor, MI, USA) connected to a pH meter, HI 99161 (Hanna instruments, Ann Arbor, MI, USA).

#### 2.4.2. Fourier Transform Infrared Spectroscopy

The spectral measurements were performed in The Laboratory of Vibrational Spectroscopy belonging to The Faculty of Chemistry at Wroclaw University of Technology. The middle-infrared spectra (4000–400 cm^−1^) were collected with the use of a Fourier transform, Bruker VERTEX 70 V vacuum spectrometer equipped with an air-cooled DTGS detector (Ettlingen, Germany). The gelatine, carrageenan or sodium alginate samples were placed on the diamond crystal of the Attenuated Total Reflection accessory. The spectral data were recorded at the resolution of 2 cm^−1^ with 64 scans collected and further elaborated using Bruker OPUS software (Bruker Optik GmbH, Ettlingen, Germany).

#### 2.4.3. Scanning Electron Microscopy

Scanning Electron Microscopy (SEM) of the gelatine, carrageenan and sodium alginate microstructure was evaluated using the EVO LS15 ZEISS Scanning Electron Microscope (Zeiss, Jena, Germany). The tested samples were cut out into smaller samples around 0.5 mm^2^ and sputtered with gold for 150 s using a sputter coater Scancoat 6 (Edwards, London, UK), which finally generated a 10 nm-thick gold layer. Each coated sample was examined using a voltage of 20 kV.

#### 2.4.4. Texture Profiling Analysis

TPA was performed at room temperature using Zwick Roell Z010, type: Z6FD1 (Zwick Roell, Ulm, Germany) equipped with a head measuring load up to 100 N. The gel samples were placed between parallel flat plate fixtures fitted to a TA.XT2 Texture Analyzer (Stable Micro Systems, Surrey, UK) interfaced with a microcomputer. All measurements were made on gels equilibrated to room temperature. The gels were compressed twice at 70% deformation and with a relaxation time of 30 s. The following parameters were quantified [39]:

- hardness: maximum force required to compress the sample
- springiness: the distance the sample was compressed during the second compression to the peak force, divided by the initial sample height, reported as a percentage
- cohesiveness: the ratio of the area under the first and second compression
- gumminess: hardness × cohesiveness
- chewiness: gumminess × springiness

#### 2.4.5. Swelling Ratio

Hydrogels were weighed and soaked in 0 (distilled water), 0.01 and 0.1M NaCl solutions. Swollen gels were removed from the swelling medium at regular intervals, dried superficially with filter paper, weighed and placed in the same bath. The swelling ratio of the hydrogels was calculated by the following equation:

(1) $$\text{SR}=\frac{W_{s}}{W_{o}}$$

where *W*_s_ is the weight of a swollen hydrogel and *W*_o_ is the weight of dried hydrogel.

#### 2.4.6. Color Measurement

The color of the gels was measured with a reflective colorimeter MINOLTA CR-400 and a CR-A33d Light Projection Tube (ø 22 mm disc, Konica Minolta, Osaka, Japan) set at C illuminant and 2° standard observer. The chroma meter was calibrated before each series of measurements using a white ceramic plate (White Calibration Plate CR-A33a, Konica Minolta, Osaka, Japan) to the following coefficients values: *Y* = 93.8, *x* = 0.3158, *y* = 0.3323. The CIELAB color scale was used to measure color: L* = 0 (black) to L* = 100 (white), -a* (greenness) to +a* (redness) and –b* (blueness) to +b* (yellowness). Color differences (ΔE*ab) between control (C gels) and samples treated with DC (G and H gels) were calculated using the equation of:

(2) $$\Delta E^{*}ab=\sqrt{{\left(L^{*}\;\text{sample }\;–\;L^{*}\;\text{control}\right)}^{2}+{\left(a^{*}\;\text{sample }\;–\;a^{*}\;\text{control}\right)}^{2}+{\left(b^{*}\;\text{sample }\;–\;b^{*}\;\text{control}\right)}^{2}}$$

### 2.5. Statistical Analysis

Each experiment was performed in triplicate. The effect of three independent categorical variables, such as the current, biopolymers and sodium chloride concentration, were evaluated. A statistical analysis was performed with univariate and multivariate Analysis Of Variance (ANOVA) using Statistica 10 (StatSoft, Kraków, Poland). The differences between the mean values were identified by the Duncan Test with a confidence level at *p* < 0.05.

## 3. Results and Discussion

The statistical analysis has shown that there was no influence of using 0.1 and 0.2% (*w/v*) NaCl on the mechanical properties, color and pH of the gels, and because of that, the addition of NaCl was omitted in the discussion. However, Król and Jarmoluk [35] suggested the flow of weak DC through the hydrosol/hydrogel layer may be used in the industry as a new decontamination method. Luo et al. [40] assumed that when electric current is applied in medium with NaCl, a variety of chemical oxidants, which caused inactivation and a bactericidal effect, are generated on the electrodes. This is the reason why the addition of NaCl is necessary and its influence was tested in all experiments.

### 3.1. pH Measurement

**Figure 2:**
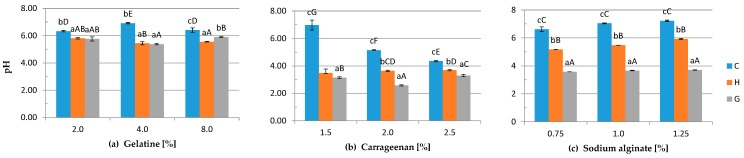
The effects of using DC on the pH of: (**a**) gelatine, (**b**) carrageenan and (**c**) sodium alginate hydrogels (C-, H-, G-types of gels) with 0.2% (*w*/*v*) NaCl and different polymer concentrations. Differences between means with the same exponent letter are not significant at a p-level value of <0.05. a–c lower case letters indicate differences between means with the same polymer concentration for different DC treatments; A–G capital letters indicate differences between means evaluated with different polymer concentrations and DC treatment.

There were significant differences between the pH in control gels and samples treated with DC (Figure 2). The pH of gelatine gels decreased from 6.91 down to 6.33 to 5.90 down to 5.38 depending on the polymer concentration. The highest values of the pH of carrageenan and sodium alginate hydrogels were measured for the C variant, lower for the H variant and the lowest for the G samples. Due to the application of DC in the hydrosol/hydrogel layer, electrolysis occurs. The splitting of water caused the changes in pH [41]. During the process, negatively-charged ions moved towards the anode and formed oxygen gas, chlorine gas, hypochlorite ions, hypochlorous acid and hydrochloric acid, while cations moved to the cathode and formed hydrogen gas and sodium hydroxide [42]. The reason why the pH of G carrageenan and sodium alginate hydrogels is lower than the pH of the H variants may be by spontaneous mixing of the solution before gelling of the H samples. During the DC application through the hydrogel layer (G samples), negatively-charged groups in the polymer network are pulled towards the positive electrode (the reaction is described in detail in Section 3.6). In accordance with the result obtained by Król and Jarmoluk [35] in the visualization of changes in the pH of gelatine gels, there is a distinct boundary between the pH on the anode and cathode side. It is possible that the area of low pH was greater and reached the geometric center of the gel where the measurement was made. This statement is in accordance with Kwon et al.’s research [43]. They observed a greater change in pH inside an anionic gel than at the cathode side. This difference was explained by the fact that positively-charged H^+^ ions generated at the anode could easily be accommodated within the negatively-charged polymer network and could influence the local pH, while the OH^-^ ions generated at the cathode were excluded from the negatively-charged gel by Donnan exclusion, which caused the smaller effect on local pH.

### 3.2. Fourier Transform Infrared Spectroscopy

**Figure 3:**
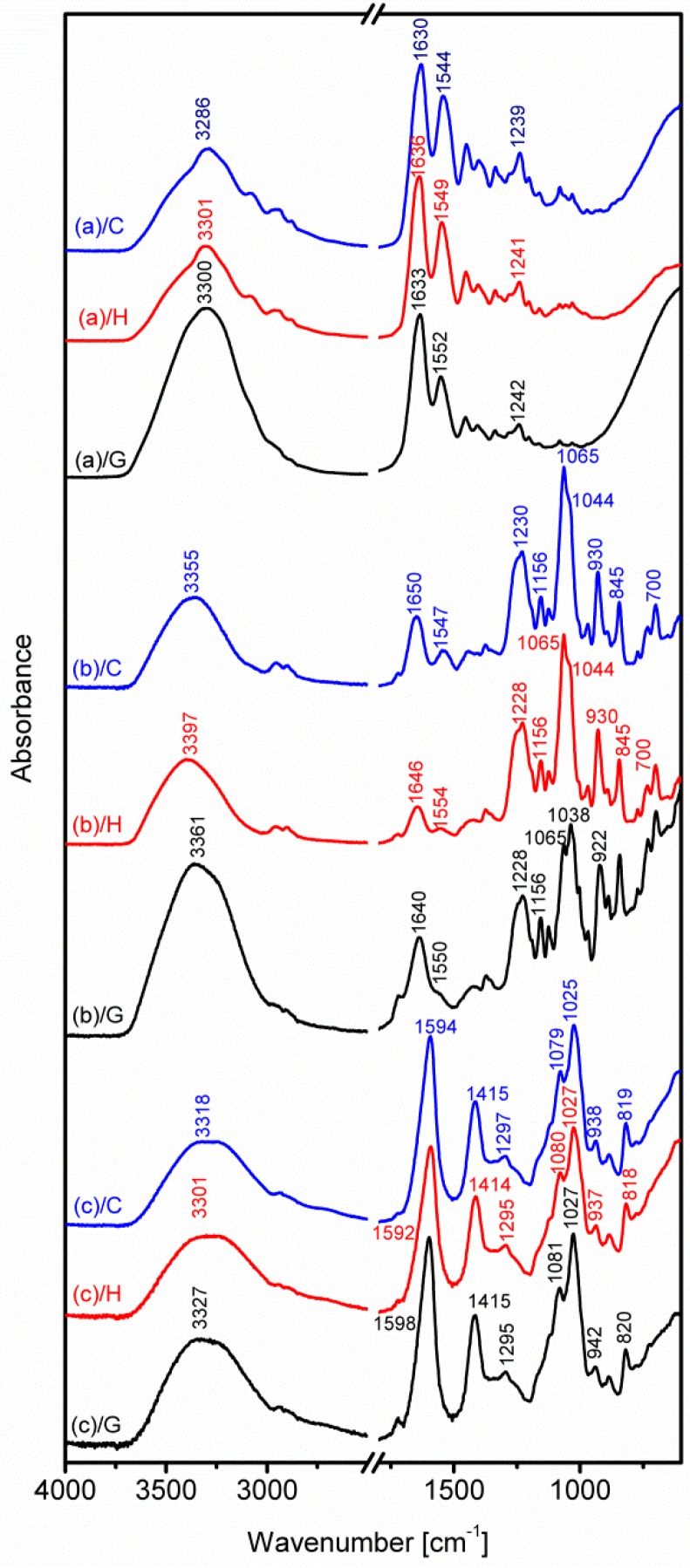
The FTIR spectra of: (**a**) 8% (*w*/*v*) gelatine + 0.2% (*w*/*v*) NaCl, (**b**) 2.5% (*w*/*v*) carrageenan + 0.2% (*w*/*v*) NaCl and (**c**) 1.25% (*w*/*v*) sodium alginate + 0.2% (*w*/*v*) NaCl in three variants of the gels: C, H, G (see the description in the text). Only discussed bands are labelled.

The main purpose of the presented IR spectroscopic research is to detect any possible changes in the hydrogel structure of (a) gelatine , (b) carrageenan and (c) sodium alginate after applying DC. The IR spectra were measured for three variants (C, H, G; see the text and Table 2) of each mentioned natural polymer. The results are collected in Figure 3 in the following order: gelatine (at the top), carrageenan (in the middle) and, at the bottom, sodium alginate.

Visual comparison of the spectra measured for the C, H and G variants demonstrates that they are very similar, except small differences in the positions of some bands.

The largest wavenumber differences are observed for intense and broad bands with maxima between 3300 and 3400 cm^−1^. These bands are mainly generated by the stretching vibrations of the O–H groups, frequently involved in hydrogen bonding, which may be the main reason for the discussed band shifts. Moreover, the possible reason of the small shifts of bands with maxima between 3300 and 3400 cm^−1^ in the discussed spectra can be due to the DC. The higher frequency of O–H stretching vibrations in G and H than in the control sample (except sodium alginate) is observed. That can indicate the weaker hydrogen bonding in the case of samples treated with DC (H and G variants) than in control samples. When the hydrogen bonding is weak or is not formed, the shifting toward higher wavenumbers (variants H, G) can be observed. DC does not change the structure of components, but they can be less crosslinked because the hydrogen bonding is responsible for the form of the structures of polymers.

In the case of gelatine, this is observed for the amide I band position, at 1630 cm^−1^ for the control sample and at 1636 cm^−1^ in the (a)/H sample spectrum. This band is characteristic for the stretching vibration of the C=O group, which often functions as an acceptor in hydrogen bonds [44,45,46]. The difference in the position of the amide II band (1552 cm^−1^) in the (a)/G spectrum with reference to the control sample (1544 cm^−1^) is 8 cm^−1^. This band is due to the N–H bending vibration and the small contribution of the C–N stretching vibration [44,45]. The former N–H groups also contribute to hydrogen bonds, which supports the conclusion that some small differences in hydrogen bond systems may mainly cause the above shifts. 

**Figure 4:**
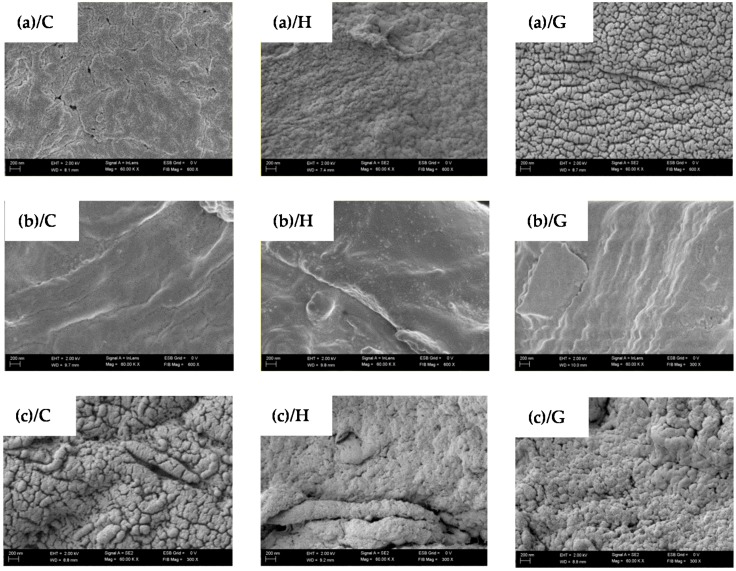
SEM images of the surface of the hydrogels of: (**a**) 8% (*w*/*v*) gelatine + 0.2% (*w*/*v*) NaCl, (**b**) 2.5% (*w*/*v*) carrageenan + 0.2% (*w*/*v*) NaCl and (**c**) 1.25% (*w*/*v*) sodium alginate + 0.2% (*w*/*v*) NaCl in three variants C, H, G (see the description in the text).

In the case of carrageenan, the strong band at 1065 cm^−1^ ((b)/C and (b)/H spectra) decreases intensity in the (b)/G spectrum. Simultaneously, the adjacent shoulder at 1044 cm^−1^ ((b)/C, (b)/H) becomes more intense in the case of (b)/G. Both absorptions are attributed to the combinations of S=O and C–O modes and may be sensitive to hydrogen bonds. Another small wavenumber difference (8 cm^−1^) is observed for the peak at 930 cm^−1^, which is assigned to the C–O stretching vibrations of 3,5-anhydro bridges [46,47]. The weak absorption at about 1550 cm^−1^ was previously not observed for different carrageenan configurations [48], and its origin here is uncertain. As is shown in Figure 4, for the sodium alginate, only two significant differences are recorded in the “fingerprint” region. The most intense band at 1594 cm^−1^, which can be assigned to the asymmetric stretching of carboxylate O–C–O vibration, changes its position by a maximum of 6 cm^−1^. A smaller deviation (4 cm^−1^) is recorded for the characteristic C–O stretching vibration of uronic acid residues [49,50,51], appearing as weak peaks at 938 and at 942 cm^−1^, respectively, in the control C and G sample spectra. It is worth noting that also for sodium alginate, the presented differences are related to the vibrations of groups that may form hydrogen bonds.

All small deviations presented above may indicate that the different procedures of the bio-polymer sample preparation can slightly affect the positions of particular IR bands, but the conservation of the number of bands and their relative intensities still supports the conclusion that the structures of gelatine, carrageenan and sodium alginate are not changed upon the applied procedures.

### 3.3. Scanning Electron Microscopy

The performed SEM analysis revealed (Figure 4) morphological changes between examined samples. The smoothest surface microstructure was observed in carrageen gels (b) in contrast to sodium alginate (c), were a clear visible microstructure was observed in control conditions (c)/C. The gelatine microstructure (a) seems to be intermediate between the examined material samples. In general, the gels that were treated with DC (G) presented the roughest microstructure when compared to the other groups. The application of DC modifies the gel composition, which was confirmed by other authors [36,52]. Moreover, the hydrosols treated with DC (H), also changed their native microstructure, however to a lesser extent than the gels.

### 3.4. Mechanical Properties

**Figure 5:**
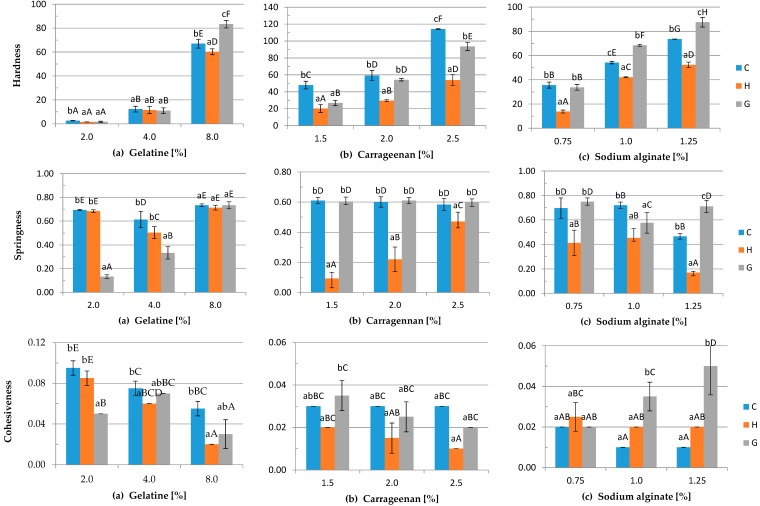

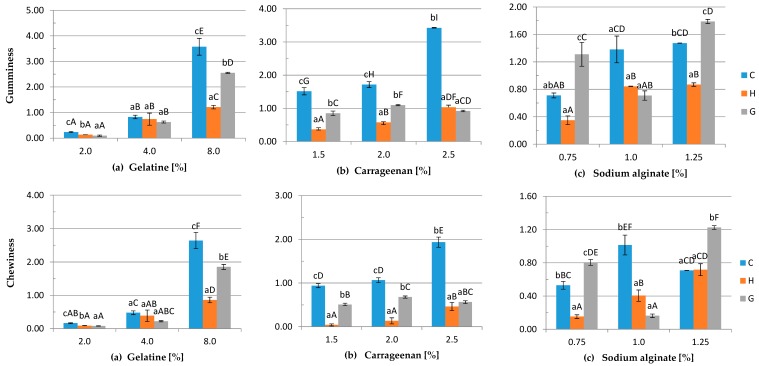
The effects of using DC on the mechanical properties of three variants of the gels: C, H, G (see description in the text) of: (**a**) gelatine, (**b**) carrageenan and (**c**) sodium alginate with different polymer concentrations and the addition of 0.2% (*w*/*v*) NaCl. Differences between means with the same exponent letter are not significant at a *p*-level value <0.05. a–c lower case letters indicate differences between means with the same polymer concentration for different DC treatments; A–I capital letters indicate differences between means evaluated with different polymer concentrations and DC treatment.

In the present study (Figure 5), it is found that the application of DC caused changes in the mechanical properties of gelatine, carrageenan and sodium alginate hydrogels. The hardness values of all of the samples increased with the concentration of polymers. The lowest hardness values were observed for H samples of carrageenan and sodium alginate for each concentration and for 8% gelatine hydrogels (60.20). Springiness (sometimes also referred to as “elasticity”) is the perception of gel “rubberiness” in the mouth and is a measure of how much the gel structure is broken down by the initial compression [53]. The lowest springiness of gelatine gels was noticed for the G hydrogel with 2% of gelatine (0.13). In the case of carrageenan and sodium alginate samples, there was a noticeable difference between the values measured for the H sample in comparison to the C and G samples. There was no significant difference between C and G samples of carrageenan, and the highest springiness was about 0.6, while the lowest (0.09) was observed for the H variant of 1.5% hydrogel. The highest cohesiveness of gelatine was measured for the control sample containing 2% of polymer (0.1), while the highest cohesiveness of carrageenan and sodium alginate was noticed for G variants of the 1.5% and 1.25% concentration of polymer, 0.04 and 0.05, respectively. Similar results were obtained for gumminess and chewiness for each tested polymer. The highest values of these parameters were measured for the C sample with 8% gelatine, the C sample with 2.5% carrageenan and the G variant with 1.25% of sodium alginate. The results obtained for mechanical properties’ measurement can be supported by the SEM micrograph depicted in Figure 4. The micrographs clearly show a morphological difference between tested samples. The G gels have the roughest microstructure, while H gels the smoothest. The reason for the difference between some of the parameters of C, H and G gels is that the application of DC may cause contractile forces to develop depending on the interplay of the various factors, including solvent polarity, pH and ionic strength. These factors are influenced by the extent of ionization of the side chains attached to the polymer backbone. The ‘squeezing effect’ of the contractile forces and electro-osmotic solvent flow modify the gel composition [36]. In our previous study, we observed noticeable partial gel mass loss and shrinkage of the gel after applying DC [35]. The study of Hsu and Block [52] demonstrated that the higher the electrical current applied, the higher the total mass lost. Moreover, although gelatine provides stable gels over a very wide range of pH values, pH should still be considered in gelatine gelation. During electrolysis, the pH of gelatine hydrosols was about 4.40 [38]. According to Pang et al. [12], gelation of gelatine can be inhibited by low pH, and because of strong electrostatic forces, which inhibit the ability of chains to form the junction zone, the gel strength is low outside the pH range 4.0–10.0. An inverse relationship is observed in the case of sodium alginate. The highest hardness was noticed for the G samples of the 1.0 and 1.25% concentration of polymer, which can be explained by the lower pH of these samples after applying DC. After electrolysis, the pH of sodium alginate G samples was about 3.58–3.71 (Figure 2). Alginate solution can form gels by lowering the pH below the pKa value of the guluronic residue (pH < 3.0) [54]. Furthermore, during the preparation of C and H hydrogels of sodium alginate, a thin layer of gel was formed on the anode, and the rest of hydrosols was used for the preparation of the experimental samples. Moreover, the poor mechanical properties of H carrageenan could be caused by the partial hydrolysis of the polysaccharide, which can occur at low pH [20].

### 3.5. Swelling Ratio

**Figure 6:**
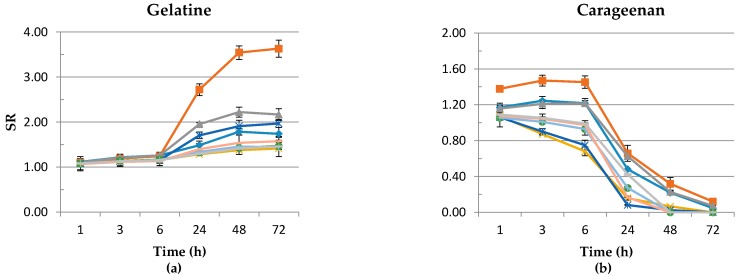

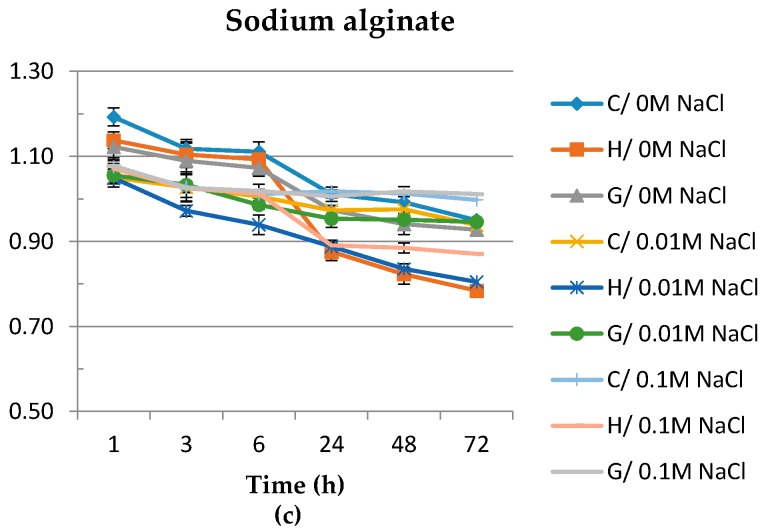
Swelling ratio of (**a**) 8% (*w*/*v*) gelatine + 0.2% (*w*/*v*) NaCl, (**b**) 2.5% (*w*/*v*) carrageenan + 0.2% (*w*/*v*) NaCl and (**c**) 1.25% (*w*/*v*) sodium alginate + 0.2% (*w*/*v*) NaCl in three variants of the gels: C, H, G (see description in the text), submerged in different NaCl concentrations.

In accordance with Haider and Park [55], we observed that after 72 h of storage, the SR of gelatine gels was higher than in the first hour (Figure 6). The highest SR in 72 hours was measured for the H/0 M NaCl variant, 3.63, while the lowest SR was observed for the C/0.01 M NaCl variant, 1.414. This results confirmed that gelatine is an effective biomaterial as wound dressings [15,16]. Similar results were obtained for carrageenan samples. The highest SR was measured for H/0 M NaCl variants at the beginning and end of storage, 1.378 and 0.119, respectively. However, the observed dissolution of the surface of carrageenan gels was slow, and it is in agreement with the results for carrageenan films obtained by Distantina et al. [56]. The researchers suggest that swelling and rapid disintegration (about 30 min) prove the hydrophilic nature of carrageenan. To decrease the solubility, carrageenan may be modified [57]. In our own study, we observed a noticeable increase of the SR of H and G gelatine and H carrageenan hydrogels after the applied DC. This is in agreement with Hsu and Block [52], who suggest that the swelling behavior of the hydrogels can be changed by electric current. After applying DC, the charge density and the electrostatic repulsion in gel networks, as well as the hydration extent of the polymer are changed [58]. The differences in the microstructure of all samples is shown in the SEM micrograph (Figure 4). The highest SR of sodium alginate samples was measured in the first hour for the C/0 M NaCl variant and was equal to 1.193, while for the H and G variants, it was lower, 1.138 and 1.122, respectively. This can be correlated with changes in the pH after applying DC (Figure 2). For C, H and G gels of 1.25% sodium alginate, the pH was equal to 7.23, 5.92 and 3.71, respectively. According to Hezaveh and Muhamed [59], the lower swelling of H and G samples can be due to the protonation of carboxylic groups and the creation of more hydrogen bonds in sodium alginate hydroxyl groups, resulting in more compact networks and, therefore, less swelling. A more compact network can explain the highest hardness of the G sodium alginate samples (Figure 5c). The lowest SR was measured for the C/0.01 M NaCl variant. The hydrogels tend to swell at a low NaCl concentration, and at a high salt concentration, they tend to shrink. This behavior was confirmed by other authors [60,61]. The swelling ability of hydrogels in various salt solutions is appreciably decreased compared to the swelling values in distilled water. The main reason for this undesired swelling loss is usually attributed to the “charge screening effect” of the cations, which as a consequence prevents an efficient electrostatic repulsion. Therefore, the Donnan pressure (the difference in osmotic pressure inside and outside the gel), which is the driving force for swelling, is decreased.

### 3.6. Color Measurement

The color performance of hydrogels is presented in Table 3. Significant changes in the total color difference were measured for H and G hydrogels of all tested polymers. Although the differences in color values were found using instruments, there still were no differences found by visual inspection (data not shown). The highest ΔE*ab was observed for the H variant of 4 and 8% gelatine, 6.74 and 6.91, respectively, for the H variant of 1.5% carrageenan (7.42) and for the G variant of 1.0% sodium alginate (3.94). There is no research about the impact of DC on the color of hydrogels. However, this information is important for the use of hydrogels, e.g., in the food industry. The difference in color may have been caused by changes in the hydrosol/hydrogel layer during the application of DC. Tanaka et al. [62] suggested that electric current produces a force not only on the mobile ions, but also on the immobile charged groups of the gel’s polymeric network. They observed that during the application of current through polyacrylamide gels, negatively-charged acrylic acid groups in the polymer network pulled the gel toward the positive electrode, while mobile H^+^ ions migrate toward the cathode. The pull creates a uniaxial stress along the gel axis with the maximum at the anode and the minimum at the cathode [1]. The pull of the polymer network towards one of the electrodes may be one of the reasons for the changes in the hydrogels’ color after applying DC. Moreover, syneresis [52], partial hydrolysis or gelling on the electrode also could cause differences in ΔE*ab.

## 4. Conclusion

Our previous study revealed that using weak DC (10–30 mA) for a short time on inoculated agar plates inhibits the growth of *S. aureus* and *Y. enterocolitica.* The authors believe that thanks to using DC, it is possible to produce innovative materials that can be used in industry, e.g., in the co-extrusion coating process, which is a promising method for laminar sanitization.

To evaluate the effects of using DC on the physicochemical properties of hydrogels and, thus, their applicability in various industries, a series of analyses was done. The results have shown that depending on whether the current is applied, in the hydrosol (C variant) or hydrogel (G variant) layer, it is possible to obtain materials with different physicochemical properties. The application of electric current in the hydrosols’/hydrogels’ layer changed their mechanical properties, swelling behavior, pH and color. However, the spectral similarity of FTIR analysis showed that the application of DC does not influence the gelatine, carrageenan and sodium alginate hydrogels’ structures. Gels exhibiting a phase transition in response to the change in external conditions, such as pH, ionic strength and electric current, are known as “stimuli-responsive” or “smart” gels, and with the newly-discovered antibacterial activity, they can find a variety of applications.

## Figures and Tables

**Table 1:** The composition of the hydrosols.

Gelatine (%)	Carrageenan (%)	Sodium alginate (%)	NaCl (%)
2.0	1.5	0.75	0.0
			0.1
			0.2
4.0	2.0	1.0	0.0
			0.1
			0.2
8.0	2.5	1.25	0.0
			0.1
			0.2

**Table 2:** Types of gels.

Run code letter	Type of gels
C	control
H	gels on the basis of hydrosols treated with DC
G	gels treated with DC

**Table 3:** The effects of DC on the color of hydrogels.

Polymer	Concentration of polymer (%)	Type of gel	L *	a *	b *	ΔE*ab
**Gelatine**	2	C	50.66 ± 0.34 ^b,c^	0.00 ± 0.00 ^a,b^	2.68 ± 0.21 ^a^	0.00 ± 0.00 ^a^
		H	52.45 ± 0.87 ^c,d,e^	0.03 ± 0.01 ^b^	2.33 ± 0.17 ^a^	3.88 ± 0.32 ^b^
		G	51.38 ± 0.52 ^c,d^	−0.06 ± 0.02 ^a^	2.41 ± 0.04 ^a^	3.23 ± 0.52 ^b^
	4	C	48.15 ± 0.21 ^a,b^	−0.07 ± 0.02 ^a^	2.86 ± 0.16 ^a,b^	0.00 ± 0.00 ^a^
		H	54.89 ± 0.31 ^e^	−0.29 ± 0.04 ^c^	2.77 ± 0.18 ^a,b^	6.75 ± 0.45 ^c^
		G	52.81 ± 0.89 ^c,d,e^	−0.02 ± 0.01 ^a,b^	3.25 ± 0.25 ^b^	2.55 ± 0.15 ^b^
	8	C	46.71 ± 0.14 ^a^	−0.43 ± 0.01 ^d^	3.82 ± 0.14 ^c^	0.00 ± 0.00 ^a^
		H	53.61 ± 0.57 ^d,e^	−0.26 ± 0.05 ^c^	4.05 ± 0.13 ^c^	6.91 ± 1.12 ^c^
		G	48.61 ± 0.97 ^a,b^	−0.50 ± 0.09 ^d^	4.67 ± 0.25 ^d^	3.73 ± 0.71 ^b^
**Carrageenan**	1.5	C	47.76 ± 1.12 ^a,b^	0.10 ± 0.07 ^a,b,c^	3.20 ± 0.16 ^e^	0.00 ± 0.00 ^a^
		H	55.07 ± 0.65 ^d^	0.08 ± 0.01 ^a,b^	1.95 ± 0.18 ^a^	7.42 ± 1.24 ^d^
		G	52.09 ± 0.32 ^c^	0.04 ± 0.02 ^a^	2.44 ± 0.22 ^b^	4.59 ± 0.60 ^c^
	2.0	C	48.31 ± 0.45 ^a,c^	0.12 ± 0.03 ^a,b,c^	3.71 ± 0.15 ^c^	0.00 ± 0.00 ^a^
		H	50.16 ± 0.87 ^b,c^	0.11 ± 0.03 ^a,b,c^	2.49 ± 0.21 ^b^	2.57 ± 0.35 ^b^
		G	49.97 ± 0.45 ^b,c^	0.11 ± 0.02 ^a,b,c^	3.77 ± 0.17 ^b^	1.95 ± 0.18 ^b^
	2.5	C	46.80 ± 0.63 ^a^	0.17 ± 0.05 ^b,c^	4.24 ± 0.13 ^c^	0.00 ± 0.00 ^a^
		H	51.07 ± 0.31 ^c^	0.19 ± 0.04 ^d^	2.61 ± 0.06 ^b^	4.88 ± 0.57 ^c^
		G	48.02 ± 0.65 ^a,b^	0.12 ± 0.02 ^a,b,c^	3.83 ± 0.13 ^c,d^	1.83 ± 0.32 ^b^
**Sodium alginate**	0.75	C	47.08 ± 0.97 ^b,c^	0.20 ± 0.08 ^a,b^	2.90 ± 0.25 ^b,c,d^	0.00 ± 0.00 ^a^
		H	50.29 ± 1.36 ^d^	0.18 ± 0.06 ^a,b^	3.50 ± 0.16 ^d,e^	3.29 ± 1.01 ^c^
		G	48.43 ± 0.82 ^c,d^	0.20 ± 0.02 ^a,b^	2.46 ± 0.09 ^a,b,c^	1.42 ± 0.57 ^b^
	1.0	C	44.73 ± 0.38 ^a,b^	0.32 ± 0.05 ^c,d^	2.33 ± 0.21 ^a,b^	0.00 ± 0.00 ^a^
		H	46.00 ± 0.46 ^a,b,c^	0.27 ± 0.03 ^b,c^	3.22 ± 0.19 ^c,d,e^	3.54 ± 0.16 ^c,d^
		G	46.74 ± 0.73 ^a,b,c^	0.26 ± 0.06 ^b,c^	2.11 ± 0.15 ^a,b^	3.94 ± 0.19 ^d^
	1.25	C	43.85 ± 0.32 ^a^	0.18 ± 0.01 ^a,b^	2.90 ± 0.31 ^b,c,d^	0.00 ± 0.00 ^a^
		H	46.10 ± 0.58 ^a,b,c^	0.15 ± 0.02 ^a^	3.81 ± 0.07 ^e^	2.90 ± 0.14 ^c^
		G	45.38 ± 0.61 ^a,b,c^	0.40 ± 0.12 ^d^	1.93 ± 0.12 ^a^	3.66 ± 0.09 ^c,d^

^a^^–e^ Different superscript letters in the same column are significantly different (*p* ≤ 0.05) according to ANOVA.
